# Biogenic synthesis of reduced graphene oxide from *Ziziphus spina-christi* (Christ’s thorn jujube) extracts for catalytic, antimicrobial, and antioxidant potentialities

**DOI:** 10.1007/s11356-022-21871-x

**Published:** 2022-07-20

**Authors:** Alaa El Din Mahmoud, Nourhan El-Maghrabi, Mohamed Hosny, Manal Fawzy

**Affiliations:** 1 Environmental Sciences Department, Faculty of Science, Alexandria University, Alexandria, 21511 Egypt; 2 Green Technology Group, Faculty of Science, Alexandria University, Alexandria, 21511 Egypt; 3 National Egyptian Biotechnology Experts Network, National Egyptian Academy for Scientific Research and Technology, Cairo, Egypt

**Keywords:** Reduced graphene oxide, Biogenic synthesis, Environmental application, Mechanism, Medicinal application

## Abstract

In the current work, various concentrations of the aqueous extract of *Ziziphus spina-christi* were employed for the phytoreduction of graphene oxide (GO). The green synthesized reduced graphene oxide (rGO) was characterized through UV-Vis spectrometry, Fourier transform infrared spectroscopy (FT-IR), scanning electron microscopy, and energy-dispersive X-ray spectroscopy (SEM-EDX). Gas chromatography-mass spectrometry (GC-MS) denoted the presence of numerous phytoconstituents including ketones, terpenoids, fatty acids, esters, and flavonoids, which acted as reducing and capping agents. The obtained results indicated the increase in rGO yield and shape with increasing the extract concentration. The optimized rGO was instantaneously ~100% removed methylene blue (MB) from the water at 5 mg L^−1^. However, the removal efficiency was slightly declined to reach 73.55 and 65.1% at 10 and 15 mg L^−1^, respectively. A powerful antibacterial activity for rGO particularly against gram-negative bacteria with a high concentration of 2 × 10^8^ CFU mL^−1^ was confirmed. Furthermore, rGO demonstrated promising and comparable antioxidant efficiency with vitamin C against DPPH free radical scavenging. While vitamin C recorded 13.45 and 48.4%, the optimized rGO attained 13.30 and 45.20% at 12 and 50 μg mL^−1^, respectively.

**Supplementary Information:**

The online version contains supplementary material available at 10.1007/s11356-022-21871-x.

## Introduction

Graphene is the basic structure of carbon materials (Allen et al. 2010). It is a noteworthy material due to its astonishing properties. It is the thinnest and strongest material on earth as well as possesses high electrical conductivity and great optical properties (Mahmoud et al. 2018b; Szőri et al. 2013). One of the exceptional properties of graphene is that it can be manipulated with other elements and metals to produce different materials with new superior properties (Radamson 2017).

Such unique properties made graphene potentially useful in a broad range of applications for environmental, medicinal, and energy issues such as energy-electrical conversion (Weng et al. 2019), fuel cells (Farooqui et al. 2018), solar-thermal conversion (Wu et al. 2019), photovoltaics (Das et al. 2019), photocatalysis (Raizada et al. 2019), water treatment (Mahmoud et al. 2020), desalination (Homaeigohar and Elbahri 2017), gas adsorption (Szczęśniak et al. 2017), biosensors (Jiang et al. 2020; Mousazadeh et al. 2021), gas sensors (Stanford et al. 2019), light-emitting diodes (Chen et al. 2018), laser (Wang et al. 2018), transistors (Kireev et al. 2017), tissue engineering (Bai et al. 2018), imaging (Campbell et al. 2019), capacitors (Anandhi et al. 2022; Correas-Serrano et al. 2018), membranes (Mi 2019), conductive inks (Karagiannidis et al. 2017), high-frequency electronics, and flexible electrodes (Aliprandi et al. 2017). However, the main constrain that limits its use is the complicated scale-up production systems (Wang et al. 2019).

Reduced graphene oxide (rGO) is produced by chemical, physical, or thermal reduction routes. Chemical routes require the usage of variety toxic reducing reagents (Saratale et al. 2018) such as hydroxylamine (NH_2_OH), hydrazine (N_2_H_4_.H_2_O), hydroquinone (C_6_H_4_(OH)_2_), sodium borohydride (NaBH_4_), and hydrogen sulfide (H_2_S) which are costly and not sustainable. In addition to the presence of impurities in the final product due to using these hazardous chemicals (Mahmoud et al. 2018a). These issues reflect in the easily self-aggregates of the produced graphene and the limited scalability (Agudosi et al. 2020). Another route is the thermal reduction of GO which was applicable in a simple way by applying heat (thermal annealing reduction) (Xiang et al. 2022). However, it is not preferable for the mass production of graphene compared to other reduction routes which can be conducted at room temperature or slightly elevated temperature (Jiříčková et al. 2022).

Biological methods include the use of either microorganisms or plants. The use of plant extracts, in particular, for the synthesis of rGO is of high interest as it is simple, safe, cost effective, non-toxic and gives higher yield than using bacteria and fungi (Mahmoud 2020b). Plants’ great potential in graphene synthesis is due to the wide variety of effective phytochemicals such as flavones, ketones, amides, terpenoids, phenols carboxylic acids, proteins, coenzymes, and carbohydrates that are able to effectively reduce graphene oxide into rGO (Verma and Chandel 2019). Various plant extracts were used to phytosynthesized rGO with the purpose of different applications in environment and medicine (Akhavan et al. 2014), for instance, leaf extract of *eucalyptus* species and rGO application in dye removal (Jin et al. 2018), *Cannabis sativa L*, *Punica granatum L*, and *Phoenix dactylifera* to evaluate their reduction activity in preparation of rGO (Ousaleh et al. 2020), fruit of *Phyllanthus emblica* for rGO in supercapacitor application (Madhuri et al. 2021), bark extract of *Alstonia scholaris* and rGO application anionic and cationic dyes decontamination (Ghosh et al. 2021), and green tea polyphenols and rGO application in cancer therapy (Akhavan et al. 2012). However, no literature is available on using the leaf extract of *Ziziphus spina-christi* for reducing graphene oxide.

In this work, *Ziziphus spina-christi* leaf extract was employed as a green reducing agent to synthesize graphene oxide. This is a further step toward the empowerment of green chemistry approach. The objectives of this work are to test the reduction capability of *Ziziphus spina-christi* extract to reduce graphene oxide and depict the influence of plant extract on the shape and yield of the synthesized rGO. Furthermore, the catalytic, antimicrobial, and antioxidant potentialities of the optimum synthesized rGO were evaluated.

## Materials and methods

### Chemicals and materials

All chemicals used without further purification in this work comprising graphite powder, potassium permanganate (KMnO_4_), hydrogen peroxide (H_2_O_2_), sodium nitrate (NaNO_3_), sulfuric acid (H_2_SO_4_), sodium borohydride (NaBH_4_), sodium hydroxide (NaOH), and methylene blue (MB) were purchased from Merck, USA.

### Preparation of *Ziziphus spina-christi* extracts

The collected leaves of *Ziziphus spina-christi* (Zi) were firstly collected from Alexandria city in Egypt. They were then dissected, washed, and rinsed using water and double distilled water (DI). The leaves were then oven dried for 72 h at 60 °C. The dried leaves were grounded in a stainless steel mixer to get fine powder.

In total, 500, 2500, and 5000 mg of Zi biomass were added to 100 mL of DI representing concentrations of 5, 25, and 50 mg mL^−1^, respectively. Each mixture was stirred at room temperature for 90 min with a stirring rate of 400 rpm (magnetic stirrer; FALC, F91T, Italy) then filtered using Whatman 8-μm filter paper, and the filtered solution is kept at 4.0 °C for further usage.

### Synthesis of reduced graphene oxide

Graphene oxide (GO) was synthesized adopting the modified Hummer method according to our previous work (Mahmoud et al. 2022). The GO solution (1 mg mL^−1^) was sonicated for 1 h until a brownish color homogeneous dispersion was gained. Fifty milliliters of *Zi* extract was added to 50 mL of GO then the mixture was stirred and heated at 70 °C for 12 h. Afterwards, a black colored solution was obtained then centrifuged at 5000 rpm and washed three times with DI. Furthermore, the washed solution was oven dried at 60 °C overnight to get dry rGO.

### Characterization

UV-Vis spectroscopy was analyzed for 1 mg mL^−1^ of GO and rGO suspensions. Subsequent to the preparation of the suspensions, they were diluted to assure translucency prior of the measurements using PG Ltd, UK. A scanning electron microscope (SEM; JOEL-JSM-IT200) with an energy dispersive X-ray spectroscope (EDX) was utilized to examine the surface morphologies and elemental composition of the samples. The prepared samples for SEM were coated with gold using ion sputter evaporator (JFC-1100E-JOEL). Fourier transform infrared (FT-IR) spectra were measured by Cary 630 (Agilent Technologies, Germany) with attenuated total reflectance (ATR) at 4 cm^−1^ resolution. An X-ray diffractometer (Brucker D2 Phaser, Germany; 5°–100° range and the rate of scanning = 5° min^−1^) was used to provide information on the crystallite structure. The d-spacing (interlayer distance) was computed using the Bragg equation (eq. 1). In addition, the phytoconstituents of *Zi* extract were identified using gas chromatography-mass spectrometry (GC-MS; Thermo Scientific, USA). Details of the procedure can be found in Hosny et al. (2021). The measurements were performed three times to get replicate results and the identified constituents were compared according to their retention time and mass spectra with the database of WILEY 09 and NIST 11.

1 $$d=\frac{\lambda }{2\ \mathit{\sin}\uptheta},$$

where λ = 0.154 nm, θ = the angle of diffraction.

### Catalytic degradation of methylene blue (MB)

0.1 mL of the optimized rGO was added to 10 mL of various concentrations of MB ranging from 5 to 15 ppm (mg L^−1^) which were prepared. Then 0.1 mL of the optimized rGO was applied with 0.1 mL of 0.06 M NaBH_4_ solution to the mixtures stirred at room temperature. The time-dependent absorption spectra of these mixes at 664 nm were used to track the degradation progress of MB. Control experiments were carried out under the identical experimental conditions in the absence of rGO and NaBH_4_. Monitoring the degradation of MB was done from 200 to 800 nm at specific time intervals at 25 °C, and it was measured by eq. 2 (Fungaro et al. 2021; Mahmoud 2020a; Mahmoud et al. 2021). The conducted experiments were done in duplicates.

2 $$\mathrm{Degradation}\ \mathrm{percentage}\%\mathrm{of}\ \mathrm{MB}=\frac{X_0-X\ }{X_0}\times 100,$$

where *X*_0_ and *X* represent the initial and final absorbance of MB, respectively.

### Antimicrobial test

The strains of gram-negative bacteria (*Escherichia coli*, *Klebsiella pneumonia*) and gram-positive bacteria (*Bacillus subtilis*, *Staphyllococus aureus* (Mrsa)) were chosen in this work. The inoculum was prepared onto tryptic soy agar plates where the reference culture strain was subcultured in glycerol broth. Following overnight incubation, 3–5 colonies of pure culture were examined with *Escherichia coli* (ATCC 8739), *Klebsiella pneumonia* (ATCC 1388), *Bacillus subtilis* (ATCC 6633), and *Staphyllococus aureus* (Mrsa) (ATCC 25923) where they were suspended in sterile test tube containing 2 mL saline.

The density of the organism suspension was modified by adding either bacteria or sterile saline, and the turbidity of the suspended colonies was compared to the 0.5 McFarland turbidity standard (2 × 10^8^ CFU mL^−1^).

Muller seeded agar was weighed and dissolved in DI before being divided into 25 mL in six flasks and autoclaved. After cooling to 50 °C, tested reference strains (1%) are introduced to sterile agar. Shaken flasks were emptied into sterilized petri dishes and allowed to set. Each seeded agar plate has three wells (each 8-mm diameter) drilled with a sterile cork borer. After sterilization by filtration, the panel of rGO was deposited on the infected plates using a sterile automatic pipette straight into its designated well; the plates were then stored in the refrigerator overnight to allow rGO diffusion. Subsequently, the plates’ incubation, which took 24 h, was carried out at 35±2 °C. The back of each Petri dish was viewed few centimeters on an unreflective surface and lightened with visible light to record the visual observations.

### Antioxidant activity of rGO

The activity of the free radical scavenging was tested using 2, 2-diphenyl-1-picrylhydrazyl (DPPH) assay in order to measure antioxidant efficiency of rGO sample. Triplicates of the assay were performed. Then, 1 mL of rGO sample was combined with 1 mL DPPH with a concentration of 0.2 mM which has been mixed together for 3 min in the absence of light along with DPPH control, which contains no nanoparticles.

The reduction in absorbance % of the mixture at 517 nm wavelength after 20 min is used to determine the quantity of radical compared to vitamin C (ascorbic acid) as a reference and the following equation was used for scavenging activity calculation.

3 $$\mathrm{Radical}\ \mathrm{scavenging}\ \mathrm{activity}\%=\frac{\left(\ Control\ abs.- sample\ abs.\right)}{control\ abs.}\times 100,$$

where control abs. is the measured absorbance without antioxidants and sample abs. is the measured absorbance with antioxidants (rGO or ascorbic acid) at 517 nm.

## Results and discussion

### Characterization

**Fig. 1:**
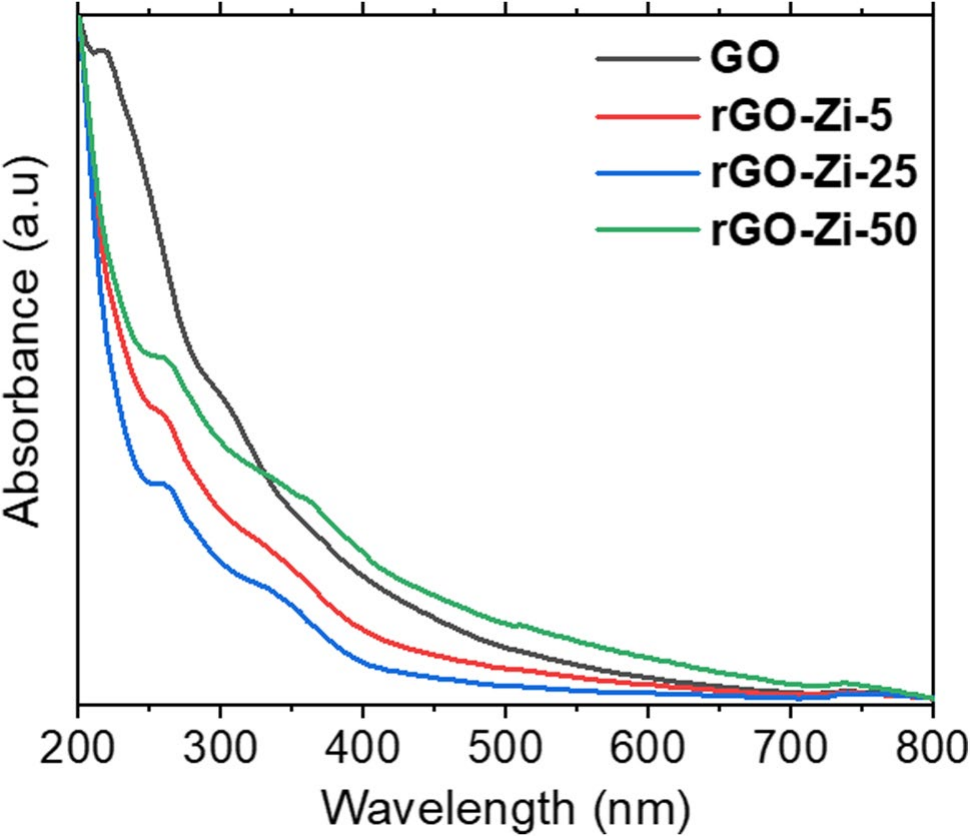
UV-Vis spectra of GO and rGO with different concentrations of *Ziziphus spina-christi* (Zi) leaf extract

The reduction of GO has been investigated using various extract concentrations of *Ziziphus spina-christi* at temperature 70 °C. The selected temperature was based on the maximum yield peak of rGO where the reduction process can occur when temperature is less than 100 °C. Figure 1 displays the effect of extract concentrations of *Ziziphus spina-christi* on the yield (absorbance peak) of rGOs. rGO yield increased to 270 nm with increasing the extract concentration subsequent to the GO reduction. This indicated the restored sp^2^ network of graphene. Furthermore, the small shoulder of GO at 335 nm disappeared in rGO samples. A similar observation is reported using other plant extracts (Ghosh et al. 2021). Ding et al. (2011) mentioned the red shift of the absorption band at 230 to 260 nm and the disappearance of the 300-nm band as an indication for the successful green synthesis of rGO nanosheets. In addition, Jin et al. (2018) observed that by increasing the concentration of *Eucalyptus* leaf extract, which was used as a reducing agent, the UV peak of the phytosynthesized rGO was red shifted to 273.5 nm. Such a result could be interpreted by the strong interaction between aromatic phytoconstituents of the leaf extract and the *π-π* bond in rGO (Wang et al. 2011).

**Fig. 2:**
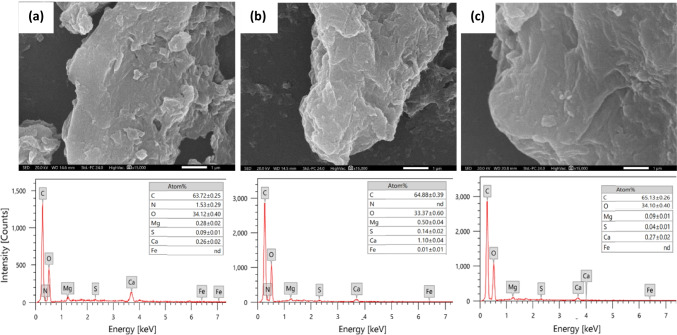
SEM micrographs and EDX of reduced graphene oxide (rGO) with **a** 5 mg mL^−1^, **b** 25 mg mL^−1^, and **c** 50 mg mL^−1^ *Ziziphus spina-christi* leaf extract

The morphological appearance of the synthesized rGOs is displayed in Fig. 2. The concentration of the plant extract slightly affected the surface morphology of rGOs. Figure 2 b and c show stacked layers with better restored surface than Fig. 2 a because of the removal of oxygen groups. However, it is noted that rGO surface was slightly corrugated and wrinkled. This may be due to the phytochemical constituents of the plant extract. Jin et al. (2018) observed the similar behavior when GO is reduced by *Eucalyptus* leaf extract.

The elemental composition of rGOs was confirmed using EDX and is illustrated in Fig. 2. The spectra show the existence of C and O elements where the ratio of O:C of the rGOs was dramatically decreased than graphene oxide after the reduction procedure as follows: rGO-Zi-5 (0.54), rGO-Zi-25 (0.51), and rGO-Zi-50 (0.52). Additionally, it revealed the absence of any impurities in the prepared samples.

**Fig. 3:**
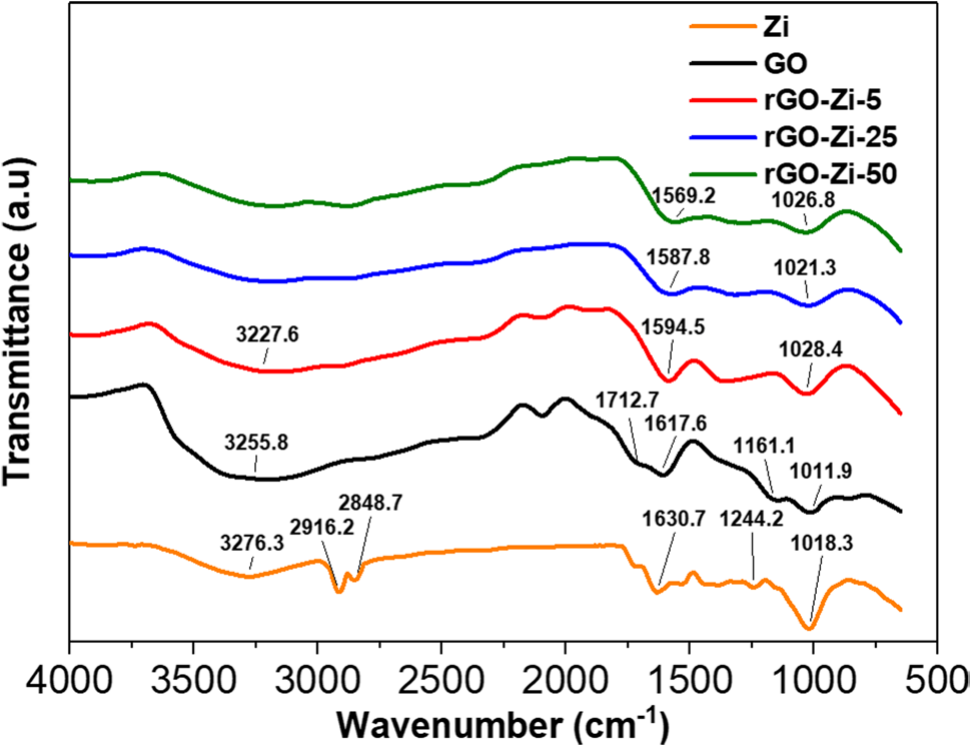
FT-IR spectra of *Ziziphus spina-christi* leaves (Zi), graphene oxide (GO), and reduced graphene oxide (rGO) using 5, 25, and 50 mg mL^−1^ of *Ziziphus spina-christi* leaf extract

The vibrational spectra of the raw and the prepared samples are illustrated in Fig. 3 to prove the role of the Zi extract as a reductant and capping agent. −OH group appeared at 3276.3 cm^−1^ and 3255.8 cm^−1^ in the spectra of Zi and GO, respectively, then its intensity decreased in rGO-Zi-5 sample and completely disappeared in rGO-Zi-25 and rGO-Zi-50 confirming the reduction of GO and this result is concomitant with Coros et al. (2020).

C–H stretch band appeared at 2916 and 2848 cm^−1^ only in the spectrum of Zi. Carboxylic acid (C=O) band group existed in GO sample at 1712 cm^−1^. Subsequent to reduction, the peak at 1712 cm^−1^ was diminished with rGOs, demonstrating decomposition of the carboxyl groups after the reduction of GO with plant extracts. Chen et al. (2014) detected different functional groups on GO such as O-H, C=O, C=C, C-OH, and C-O bonds.

It is worth noting that C=C group showed at 1630 cm^−1^ in the case of Zi but it was at 1617 cm^−1^ in GO sample which shifted to lower wavenumbers and their intensity decreased in the rGO samples. Such behavior confirmed sp^2^ carbon network restoration as indicated in Johra and Jung (2015) and Raja et al. (2021) who detected small intense peak position at 1555 cm^−1^ in rGO. Furthermore, ether group (C-O) at 1161.1 cm^−1^ is not detected in the spectra of rGOs compared to GO spectrum. Even the epoxy group (C-O) intensities that appeared in GO spectrum at 1011.9 cm^−1^ decreased especially in rGO-Zi-25 and rGO-Zi-50 samples confirming the reduction of GO.

The results of the current investigation are compatible with other literature such as Nhlane et al. (2021) and Huang et al. (2019). As a result, it was possible to deduce that the phytoconstituents found in Zi’s aqueous extract were responsible for the reduction of GO into rGO. Thus indicating the potential use of *Zi* extract as an alternative and sustainable way for rGO synthesis.

The diffraction peak of GO was detected at 2*θ* = 11°; (111) plane which corresponds to d-spacing of 0.80 nm (Fig. S1). This finding is consistent with Tambe (2022) who reported that the d-spacing of GOs synthesized with Hummers method and with additional KMnO_4_ were 0.71 and 0.86 nm, respectively. Subsequent to reduction with different plant extract concentrations, rGO-Zi-5 showed a broad peak at 2*θ* = 16.5° which corresponds as a shifting peak from GO sample. Similar behavior of such peak was detected in GO prepared by Aliyev et al. (2019), Gupta et al. (2017), Yogesh et al. (2020) where the peak position differs from 11° to 17° according to the amount of absorbed water. Besides, a sharp peak was observed at 2*θ* = 29° with d-spacing of 0.31 nm which revealed the reduction of the GO.

With increasing the plant extract concentration (Fig. S1), the peak at 2*θ* = 16.5° was almost decreased in intensity with broading peaks at 2*θ* = 28° (d-spacing of 0.32 nm) and 26° (d-spacing of 0.34 nm) corresponding to the (002) plane for rGO-Zi-25 and rGO-Zi-50, respectively. The small peak at 2*θ* = 42° revealed the successful reduction of rGO. Dominic et al. (2021) found the diffraction peak of rGO was at 2*θ* = 25° (d-spacing of 0.36 nm) that was prepared from the leaf extract of *Plectranthus amboinicus*. The high value of d-spacing of GO rather than rGO reflects the existence of water molecules and the oxygen functional groups (Yang et al. 2021). It is noteworthy that rGO peaks became broading and the values of d-spacing were different that may be due to the formations of rGO layers or sheets and restacking of graphene layers (Siddarth et al. 2019; Thakur and Karak 2012).

### rGO synthesis mechanism

**Fig. 4:**
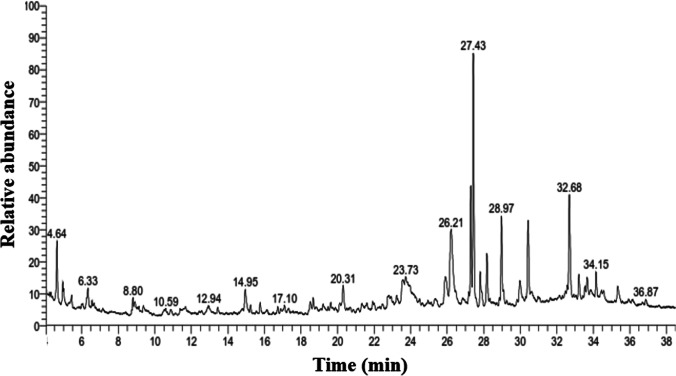
GC-MS chromatograms of *Ziziphus spina-christi* leaf extract

**Fig. 5:**
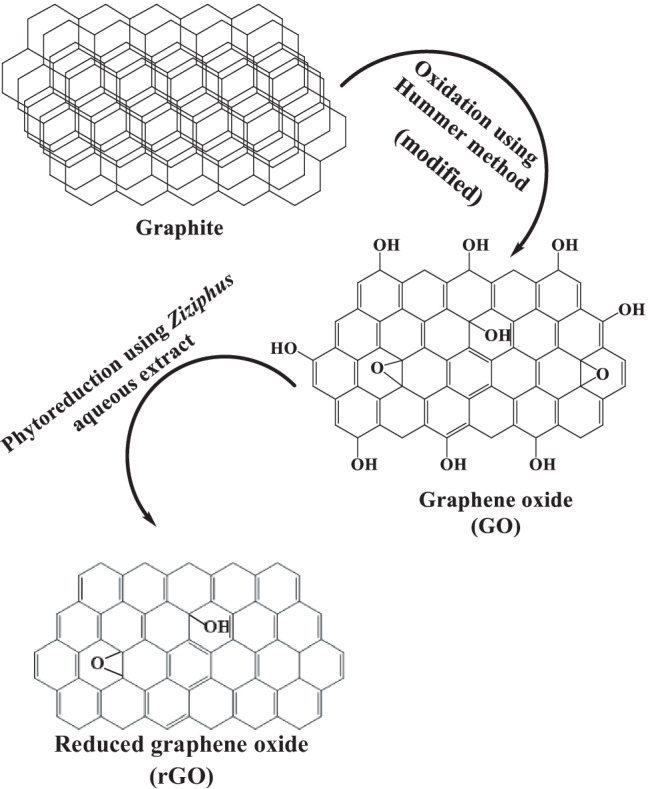
Simplified mechanism for the green synthesis of rGO using the leaf extract of *Ziziphus spina-christi*

Figure 4 illustrates the chromatogram of major compounds originating from Zi. The identified phytoconstituents may be involved in GO reduction as shown in Table 1. Ketones, terpenoids, fatty acids, esters, and flavonoids are the phytoconstituents that functioned as reducing and capping agents. It is also reported that the extract of this plant species contains flavonoids, tannins (polyphenols), and lipids (Abalaka et al. 2010). Asgarpanah and Haghighat (2012) reported that hexadecanol and ethyl octadecenoate are detected in the leaf extract of Zi. A simplified mechanism illustrating the phytofabrication of rGO *via* the aqueous extract of *Ziziphus* is presented in Fig. 5. It confirms the successful contribution of *Ziziphus* phytoconstituents in the reduction of GO into rGO.

**Table 1﻿.**
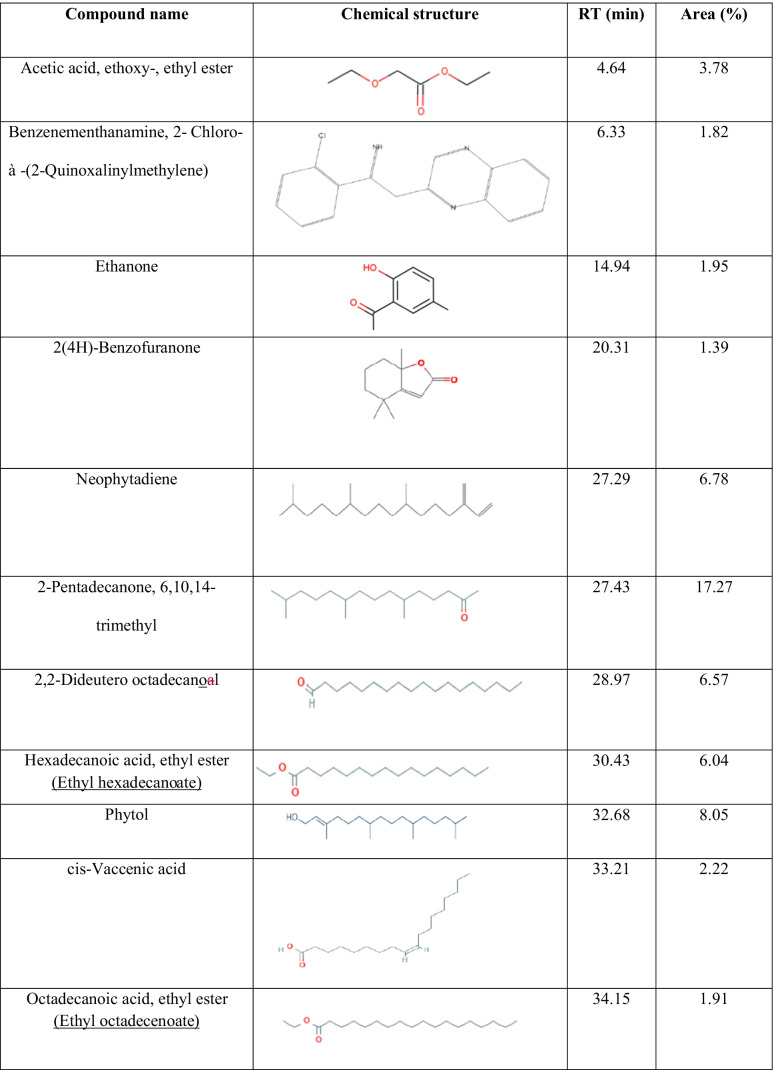
Phytoconstituents in *Ziziphus spina-christi* leaf extract using GC-MS with the retention time (RT) and the area

GO is hydrophilic due to the presence of different oxygen functional groups such as −OH and −COOH (Mahmoud et al. 2018a). The hydrophilic GO could simply be converted to relatively hydrophobic rGO and confirmed by the low ratio values of O/C in the range of 0.54–0.52 (refer to Fig. 2 and Fig. 3). The synthesized rGOs were relatively hydrophobic because of some remaining functional groups. Similar behavior was reported in Xiang et al. (2022). This could be preferable in environmental applications due to its easy separation from the aqueous solutions with centrifugation or filtration.

### Catalytic degradation of methylene blue (MB)

**Fig. 6:**
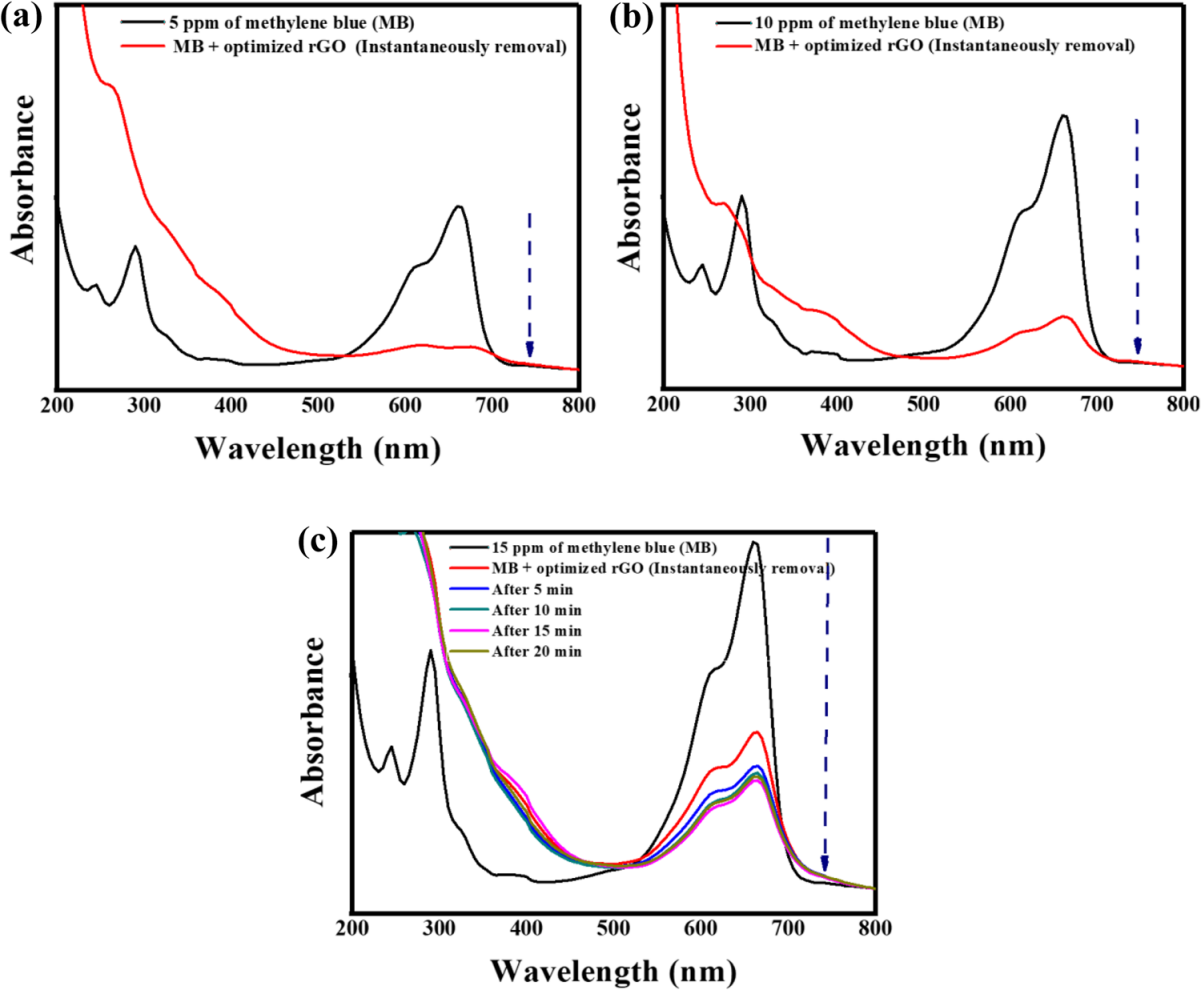
Catalytic degradation of methylene blue (MB); **a** 5 ppm, **b** 10 ppm, **c** 15 ppm using 0.1 mL of the optimized rGO

The environmental application of rGO was evaluated through the degradation of MB to leuco MB in the presence of the reducing agent NaBH_4_. 0.1 mL of green synthesized rGO with 0.1 mL of 0.06 M NaBH_4_ could degrade MB as illustrated in Fig. 6. The attained results showed the instantaneous disappearance of the blue color of 5 and 10 ppm MB.

The degradation efficiencies were recorded as ≃100% and 73.55% for 5 and 10 ppm, respectively as illustrated in Fig. 6 a and b. On the other hand, 15 ppm of MB took almost 20 min to reach a degradation efficiency of 66.53% (Fig. 6c). It is worth noting that there was no recorded degradation of MB in the control experiments conducted in the absence of rGO or NaBH_4_, showing that the green synthesized rGO is required for the catalytic degradation of MB. Keeping in mind that the international standard dye concentration in the discharged wastewater should be ˂ 1 ppm (Katheresan et al. 2018).

As NaBH_4_ is both electron donor and a prerequisite for photocatalytic degradation, an e-transmission mechanism exists between MB and NaBH_4_ via rGO where rGO successfully transported electrons from NaBH_4_ into MB. Therefore, rGO could degrade MB in short time and convert NaBH_4_ to gaseous products. Arnawtee et al. (2022) demonstrated the similar findings for photocatalytic MB degradation with multiwalled carbon nanotubes/kraft lignin/Pd nanocomposite catalyst and NaBH_4_.

When the degradation efficacy of the green synthesized rGO in this work was compared to literature, it was figured out that the synthesized rGO was significantly better in performance. CoTPP (tetramethoxyphenylporphyrin)/rGO/MWCNTs (multiwalled carbon nanotubes) nanocomposite that was prepared by Kiran et al. (2020) resulted in 50% degradation of 5 ppm MB in 70 min. Ghosh et al. (2021) showed that rGO, prepared by the bark extract of *Alstonia scholaris*, can degrade 12 ppm MB with 94.67% in 210 min. Consequently, the synthesized rGO possesses well efficiency in line with other phytosynthesized rGOs so it is thought to be a good catalyst for catalytic degradation of MB and other hazardous organic pollutants in wastewater.

**Fig. 7:**
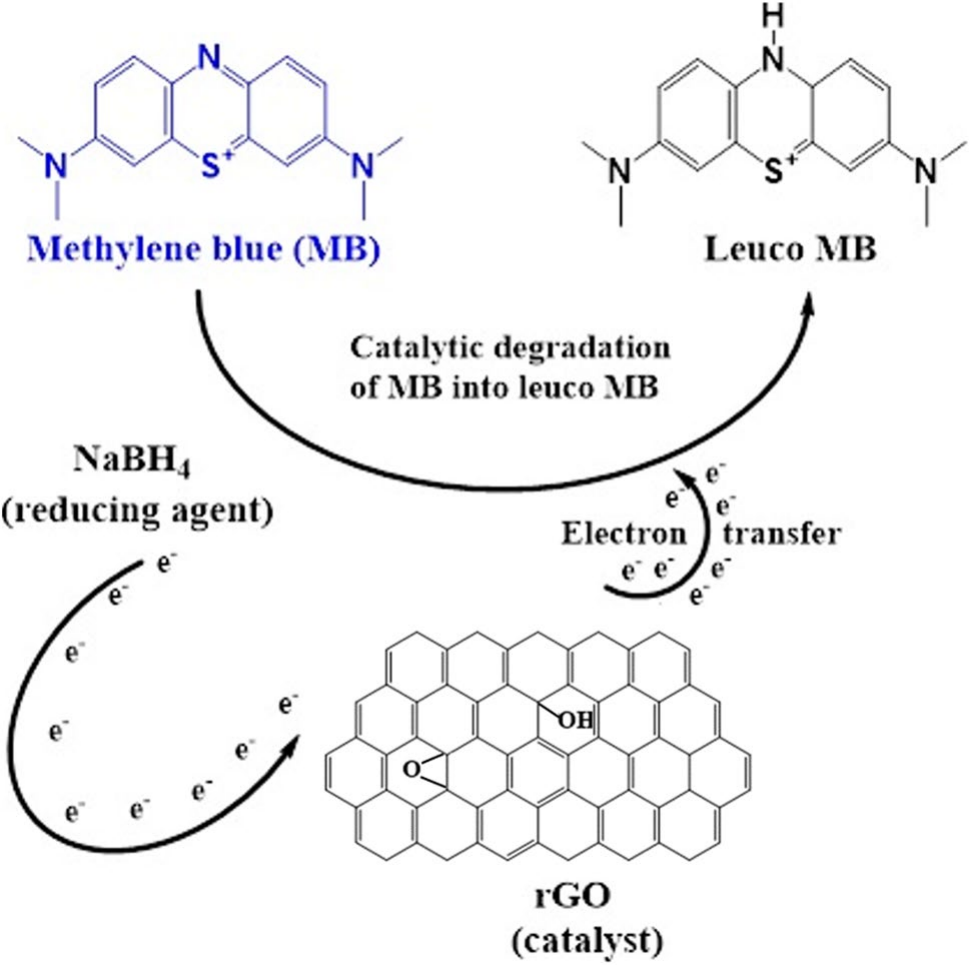
A degradation mechanism of methylene blue (MB) using the optimized rGO

Figure 7 depicts a mechanism that elucidates the active function of rGO in degrading MB into leuco MB, where rGO successfully transported electrons from NaBH_4_ into MB, resulting in its quick removal. Table 2 also provides a comparison of rGO and other nanocatalysts, highlighting key aspects in the degradation process.

**Table 2.** A comparison between the catalytic degradation efficiency of the optimized rGO and literature reported for methylene blue (MB)

Catalyst	Dye concentration (mg L^−1^)	Degradation efficiency (%)	Time (min)	Ref.
nZVI- Fe_3_O_4_/rGO.	50	98.00	60	(Yang et al. 2015)
Mn/rGO nanocomposite	50	70.40	30	(Liu et al. 2018)
Mn-Co/rGO nanocomposite		≃100		
rGO-stabilized MnO/N-doped carbon nanofibers	20	100	180	(Chen et al. 2017)
rGO/CoFe_2_O_4_	20	100	24	(Wu et al. 2016)
(rGO-Ag) nanocomposite	–	71.42	8	(Sahu et al. 2019)
MoS_2_	200	98	30	(Zou et al. 2019)
(MoS_2_-rGO) nanocomposite			10	
rGO/Fe_3_O_4_ nanocomposite	30	47.47	60	(Vinothkannan et al. 2015)
Graphene/MnO_2_ hybrids	50	≃100	5	(Qu et al. 2014)
CoTPP/rGO/MWCNTs nanocomposite	5	50.00	70	(Kiran et al. 2020)
rGO-SiW nanocomposite	35	≃100	34	(Ucar et al. 2017)
rGO	5	≃100	Instantaneously	The current work
	10	73.55		
	15	65.10		

### Antimicrobial study

The development of antimicrobial drugs is always challenging and costly (Prasad et al. 2017). Hence, nanomaterials and graphene materials may be able to fill this gap to combat the antibiotic resistance. Ahmad et al. (2020) stated that graphene-based nanomaterials demonstrated tremendous antibacterial resistance with mild cytotoxicity.

**Fig. 8:**
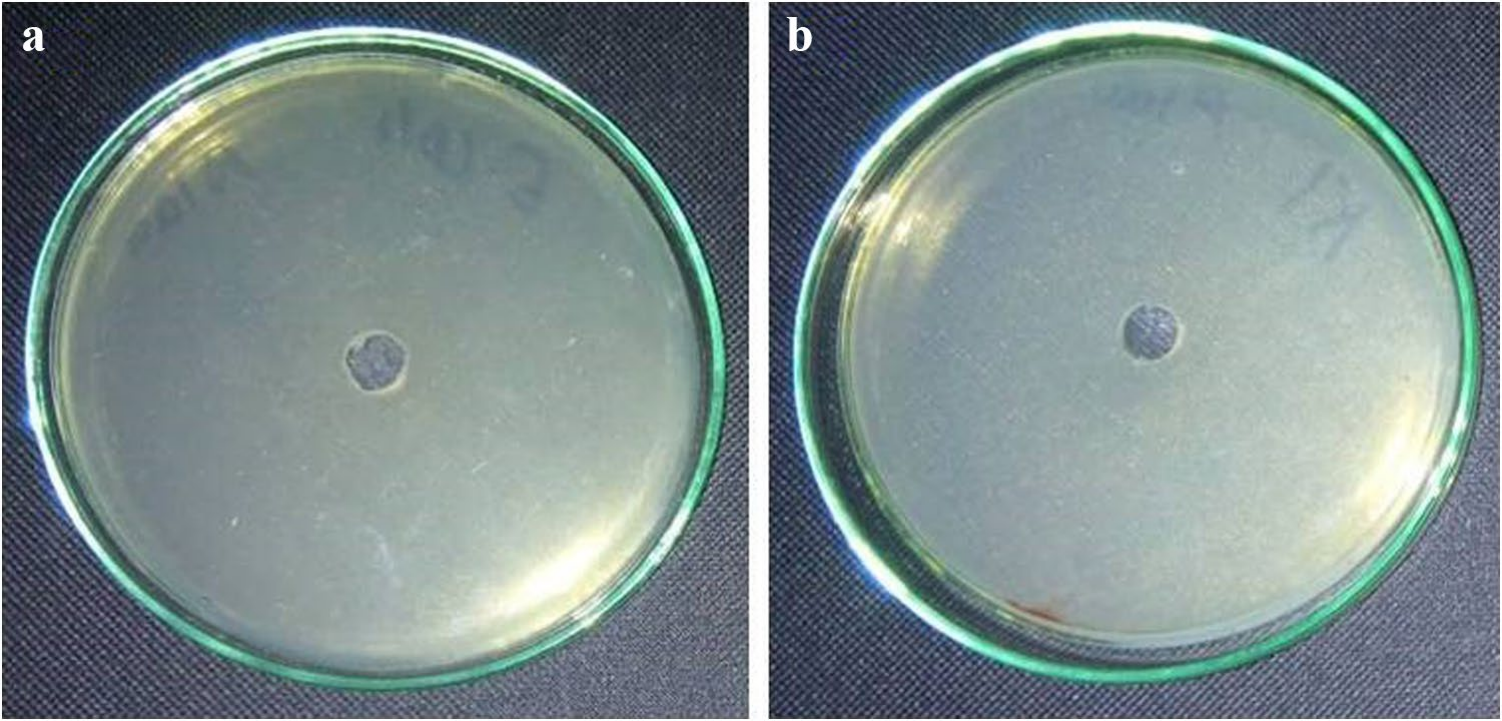
Antibacterial effect of reduced graphene oxide (rGO) against **a** *Escherichia coli* and **b** *Klebsiella pneumonia*

The efficacy of green synthesized rGO (100 mg L^−1^) as an antibacterial agent at a concentration of 100 mg L^−1^ in inhibiting various sorts of bacteria was detected by measuring the inhibition zones (Fig. 8).

Our results exhibited that there was no growth revealed in both *Escherichia coli* and *Klebsiella pneumonia* demonstrating that rGO was very effective against gram-negative bacteria. rGO, on the other hand, had no effect on gram-positive bacteria.

In literature, the *Ziziphus spina-christi* callus extract was used for the biosynthesis of SeONPs and ZnONPs then evaluated in antibacterial activity (lashin et al. 2021). Furthermore, a promising wide-spectrum antimicrobial activity was exhibited by both SeONPs and ZnONPs. However, lashin et al. (2021) added that the tested microbial strains, including *E. coli*, *Pseudomonas aeruginosa*, *S. aureus*, *B. subtilis*, *Candida albicans*, *Cryptococcus neoformans*, *Aspergillus niger*, and *Aspergillus. fumigatus* showed no inhibition zones. Recently, Chinnappa et al. (2022) reported that the composite of rGO-Ag nanoparticles have antimicrobial activity to *E. coli* with 22 mm inhibition zone.

As indicated in previous research (Liu et al. 2011; Sengupta et al. 2019), the antibacterial activity of rGO is associated to modifying the shape of the cell membrane and impeding normal budding due to a loss of membrane integrity.

Oxidative stress is an antibacterial mechanism where it is induced by the reactive oxygen species (ROS) produced by rGO or disturbing/oxidizing the cell membranes without ROS production (Liu et al. 2011). Nanomaterials could induce substantial oxidative stress resulting in DNA damage due to OH•, O2^−^, and H_2_O_2_ generation in bacterial cells leading to oxidation of polyunsaturated phospholipids (Kumar et al. 2011). Recently, the oxygen has a role in the nanobubble form with the rGO or its nanocomposite presence as a nanoshuttle that could effectively impact the cellular interactions (Jannesari et al. 2020). rGO and oxygen nanobubbles can capture electrons from the bacteria’s respiratory chain. This can be done by rGO to directly trap electrons from the cell membranes and passing the captured electrons to the O_2_ NBs for ROS formation, indirect electron capturing.

As the determined surface charge of rGO was − 24 ± 2.55 mV, there was a charge transfer between rGO and bacteria leading to antibacterial efficiency. The rGO edges could trigger a pore creation in bacterial cell wall causing osmotic imbalance and the cell death as indicated by Pham et al. (2015) even in the dark as reported in Lakshmi Prasanna and Vijayaraghavan (2015).

Herein, the possible mechanisms involved in the antibacterial activity are:

1. Direct physical connection between the rGO edges and bacterial cells can trigger physical damage to the cell membrane, resulting in disrupting cell metabolism (Akhavan and Ghaderi 2010). Liu et al. (2011) emphasized the irreversible damage of *E. coli* cells after direct contact with either GO or rGO. rGO stimulates membrane stress on bacterial cells where *E. coli* cells were embedded in rGO aggregates.
2. rGO usually leads to increasing the ROS, resulting in shrinkage and loss of cell membrane integrity, oxidative stress, impairment of DNA replication, and eventually apoptosis (Yang et al. 2019). It is found that nanoparticles were embedded with the cell membrane of bacteria by ROS (Lakshmi Prasanna and Vijayaraghavan 2015). Dutta et al. (2015) found that rGO generates ROS under visible light in air through a singlet oxygen–superoxide anion radical pathway to kill *Enterobacter* sp.
3. The bacteria could be also trapped within the aggregated rGO sheets as a kind of inactivation without any opportunity for increase in a culture medium. Further details could be referred to Akhavan et al. (2011).

As a result of the findings, rGO is a promising antibacterial with a high efficacy against gram-negative bacteria at high concentrations (2 × 10^8^ CFU mL^−1^). In addition, Table 3 shows a comparison of the antibacterial potency of rGO and other nanomaterials, demonstrating that rGO has a high antibacterial effectiveness that is superior to previously reported data.

**Table 3.** Comparison of the antibacterial efficacy of phytosynthesized rGO in the current work and that reported in literature.

Sample	Sample concentration (mg mL^−1^)	Bacterial strain	Zone of inhibition (mm)	References
rGO	–	*Bacillus subtilis*	2	(Rani et al. 2019)
		*Escherichia coli*	1.9	
rGO-Cu_2_O		*Bacillus subtilis*	3.5	
		*Escherichia coli*	3	
rGO	100	*Escherichia coli*	18	(Vatandost et al. 2020)
		*Staphyllococus aureus*	23	
GO		*Escherichia coli*	Resistant	
		*Staphyllococus aureus*	Resistant	
Pd-RGO-ZnO nanocomposite	–	*Klebsiella pneumonia*	11	(Rajeswari and Prabu 2020)
		*Pseudomonas aeruginosa*	10	
Ag-rGO nanocomposite	100	*Staphyllococus aureus*	8	(Rajeswari et al. 2017)
		*Bacillus subtilis*	9	
		*Escherichia coli*	18	
GO	100	*Bacillus subtilis*	9	(Thiyagarajulu and Arumugam 2021)
		*Escherichia coli*	8	
		*Pseudomonas aeruginosa*	6	
rGO		*Bacillus subtilis*	16	
		*Escherichia coli*	12.5	
		*Pseudomonas aeruginosa*	7.5	
rGO-ZnO nanocomposite	200	*Klebsiella pneumonia*	14	(Rajeswari and Prabu 2018)
		*Pseudomonas aeruginosa*	14.5	
GO	100	*Bacillus subtilis*	9	(Thiyagarajulu et al. 2020)
		*Escherichia coli*	8	
		*Pseudomonas aeruginosa*	6	
rGO		*Bacillus subtilis*	18	
		*Escherichia coli*	14	
		*Pseudomonas aeruginosa*	7.5	
Au-rGO nanocomposite	150	*Klebsiella pneumonia*	23.4	(Saikia et al. 2016)
		*Pseudomonas aeruginosa*	24.4	
		*Staphyllococus aureus*	21.4	
rGO	–	*Escherichia coli*	11	(Joshi et al. 2020)
rGO	100	*Escherichia coli*	No growth (sensitive)	The current work
		*Klebsiella pneumonia*	No growth (sensitive)	
		*Bacillus subtilis*	Resistant	
		*Staphyllococus aureus*		

### Antioxidant study

Byproducts as dangerous and toxic ROS are typically generated by common metabolism processes that are considered critical for the survival and protection of living organisms (Lakra et al. 2022; Saxena et al. 2022). Free radicals usually lead to oxidative stress and other health issues. DPPH is considered to be a significant and prevalent free radicals that can adversely influence human cells (Biela et al. 2022; Mohan et al. 2021). Because the free electrons are delocalized throughout the entire molecule, it is classified as a persistent free radical that is not easily degraded like the majority of other free radicals (Zhang et al. 2019). Since it is a free uncharged radical that can consume hydrogen or free electrons, DPPH has been used for many years to test the free radical capabilities of antioxidants to produce a steady diamagnetic molecule (Singh et al. 2021). The reduced form of DPPH could be created when it is mixed with a material or a nanomaterial either metallic or graphene-based materials that can give a hydrogen atom (antioxidant) which reflect an effective role against DPPH. This was demonstrated by the removal of the characteristic violet color (Flieger et al. 2021; Majumder and Gangopadhyay 2022).

**Fig. 9:**
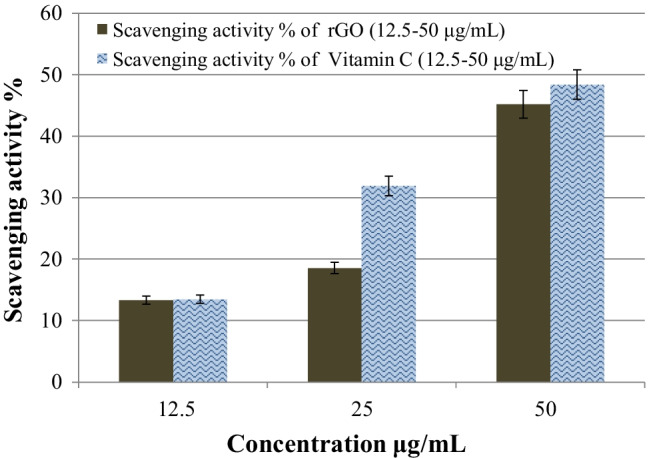
Antioxidant efficiency of reduced graphene oxide (rGO) and ascorbic acid (positive control) against DPPH

When the concentration of rGO increased from 12.5 to 50 μg mL^−1^ in the current work, the scavenging percent of DPPH grew consistently from 13.3% to roughly 45.2% (Fig. 9) which is postulated to be promising. It was found that 12.5, 25, and 50 μg mL^−1^ of vitamin C achieved 13.45, 31.9, and 48.4% of DPPH (Fig. 9**)** which are slightly higher than that of rGO. As a result, the current findings validated rGO’s excellent antioxidant capability against DPPH, as well as its potential application in the scavenging of additional free radicals in future research. Table 4 shows a comparison of rGO and other nanomaterials in terms of DPPH scavenging efficiency, demonstrating rGO’s strong antioxidant efficacy, which is concomitant with most of previously reported results. Few literature, Umekar et al. 2020, Suresh et al. 2015c, and Murugesan et al. 2018, reported higher antioxidant efficiency. However, they consumed from 3 up to 100 fold the plant concentration used herein (Table 4).

**Table 4:** Comparison of the antioxidant efficiency of rGO synthesized in the current work to graphene-based materials mentioned in literature

Antioxidant	Concentration (μg mL^−1^)	Scavenging activity (%)	References
Graphene oxide (GO)	400	40	(Baali et al. 2019)
ZnO-rGO nanocomposite		22	
(rGO)	5000	80	(Suresh et al. 2015c)
(rGO QDs) quantum dots	160	80	(Murugesan et al. 2018)
GO	5000	25	(Suresh et al. 2015b)
rGO	2000	75	
rGO	–	73.83	(Vatandost et al. 2020)
rGO-ZnO nanocomposite	200	45	(Rajeswari and Prabu 2018)
GO	200	20	(Mahmudzadeh et al. 2019)
rGO		30	
GO	750	25	(Al-Ani et al. 2019)
rGO		60	
rGO-ZnO nanocomposite	500	30	(Jafarirad et al. 2018)
(rGO-ZnO-Ag) nanocomposite		25	
rGO-ZnO-Nd nanocomposite		15	
rGO	200	25	(Rajeswari and Prabu 2020)
rGO-ZnO nanocomposite		45	
Pd-rGO-ZnO nanocomposite		55	
rGO	4000	90	(Suresh et al. 2015a)
rGO	–	25	(Umekar et al. 2020)
(rGO-TiO_2_) nanocomposite		45	
rGO	50	45.2	The current work

## Conclusion and recommendations

The adopted concentrations of the aqueous leaf extract of *Ziziphus spina-christi* were successfully utilized as a reducing and stabilizing agent in the phytoreduction of graphene oxide for the first time. SEM micrographs revealed that rGO had stacked layers with better restored surface when using the higher concentrations of plant extract, 25 mg mL^−1^. Moreover, the ratio of O:C of the synthesized rGOs were substantially diminished compared to graphene oxide after the reduction procedure as indicated by EDX. GC-MS as well as FT-IR denoted the presence of several phytoconstituents in the plant extract such as ketones, terpenoids, fatty acids, esters, and flavonoids, which are assumed to be effectively participating in rGO synthesis. Powerful catalytic degradation efficiencies ranging from 65.1 to 100% were instantaneously achieved when the optimized rGO was applied in the removal of MB with varying concentrations. Additionally, rGO exhibited a powerful antibacterial activity particularly against gram-negative bacteria with a high concentration of 2 × 10^8^ CFU mL^−1^ by inhibiting the growth of *Escherichia coli* and *Klebsiella pneumonia*. Likewise, rGO demonstrated promising antioxidant efficiency as it reached up to 98.9% compared to that of vitamin C at 12 μg mL^−1^. Consequently, it was concluded that the aqueous extract of *Ziziphus spina-christi* could be efficiently utilized in the phytosynthesis of rGO, which could be harnessed in a variety of different environmental and medical applications, in a facile, eco-friendly, and simple manner.

## Supplementary Information

ESM 1 (DOCX 31 kb)

## Data Availability

They are available upon reasonable request.
