# Supplementary material for: Biogenic synthesis of reduced graphene oxide from Ziziphus spina-christi (Christ’s thorn jujube) extracts for catalytic, antimicrobial, and antioxidant potentialities
Source: Environ Sci Pollut Res Int. 2022 Jul 20;29(59):89772–87. doi: 10.1007/s11356-022-21871-x (PMC9671977; doi:10.1007/s11356-022-21871-x)
Supplement: Supplementary file 1 — (DOCX 31 kb) [file 11356_2022_21871_MOESM1_ESM.docx]

**Supplementary information**

**Biogenic synthesis of reduced graphene oxide from *Ziziphus spina-christi* (Christ’s thorn jujube) extracts for catalytic, antimicrobial, and antioxidant potentialities**

Alaa El Din Mahmoud^1,2*^, Nourhan El-Maghrabi^1,2^, Mohamed Hosny^1,2^, Manal Fawzy^1,2,3^

^1^Environmental Sciences Department, Faculty of Science, Alexandria University, 21511, Alexandria, Egypt.

^2^Green Technology Group, Faculty of Science, Alexandria University, 21511, Alexandria, Egypt.

^3^National Egyptian Biotechnology Experts Network, National Egyptian Academy for Scientific Research and Technology, Egypt.

***Corresponding author:** Dr. Alaa El Din Mahmoud**, Email:** alaa-mahmoud@alexu.edu.eg

**Characterization**
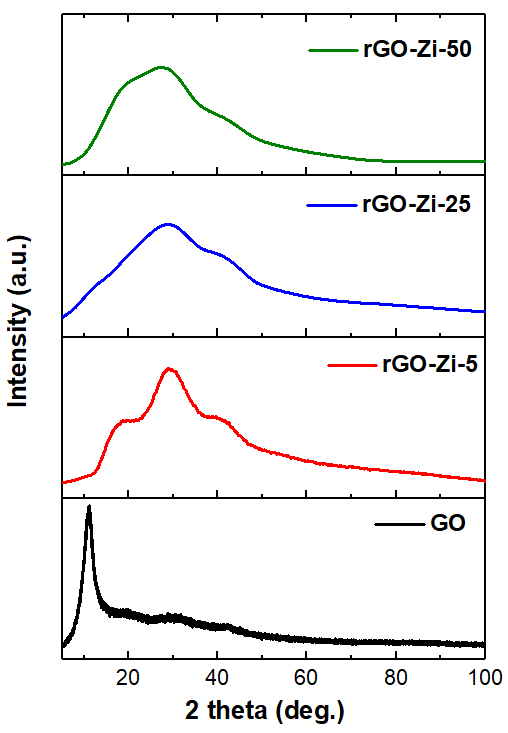


Fig. S1 XRD patterns of graphene oxide (GO) and reduced graphene oxide using 5, 25, and 50 mg mL^-1^ of *Ziziphus spina-christi* leaves extract.
